# Paper-Based Microfluidic Devices: A Powerful Strategy for Rapid Detection

**DOI:** 10.3390/mi17010064

**Published:** 2025-12-31

**Authors:** Xin Liu, Weimin Xu, Haowen Jiang, Ruping Liu, Ziqi Kong, Jianxiao Zhu, Zhicheng Sun, Shouzheng Jiao, Weiqing Li, Yang Wang

**Affiliations:** 1 School of Printing and Packaging Engineering, Beijing Institute of Graphic Communication, Beijing 102600, China; 18872843338@163.com (X.L.); weiminxu@bigc.edu.cn (W.X.); 19825484832@163.com (H.J.); kongziqi510@gmail.com (Z.K.); 15838045277@163.com (J.Z.); zhicheng@bigc.edu.cn (Z.S.); jiaoshouzheng@bigc.edu.cn (S.J.); 2 Department of Thoracic Surgery, Beijing Tiantan Hospital, Capital Medical University, Beijing 100070, China; 3 Key Laboratory of Biomechanics and Mechanobiology, Ministry of Education, Beijing Advanced Innovation Center for Biomedical Engineering, School of Engineering Medicine, Beihang University, Beijing 100191, China

**Keywords:** paper-based microfluidics, rapid detection, point-of-care diagnostics, environmental monitoring, food safety

## Abstract

In recent years, diseases, environmental pollution, and food safety issues have seriously threatened global health, generating international concern. Many existing detection strategies used to deal with the above problems have high accuracy and sensitivity, but usually rely on large-sized, complex instruments and professional technicians, which are not suitable for on-site testing. Therefore, it is imperative to develop highly sensitive, rapid, and portable analytical methods. Recently, microfluidic paper-based analytical devices (μPADs) have been recognized as a highly promising microfluidic device substrate to deal with the issues existing in medical, environmental, and food safety, etc., due to their advantages, including environmental-friendliness, high flexibility, low cost, and mature technology. This review comprehensively summarizes the recent advances in μPADs. We first overview the development of paper-based materials and their core fabrication techniques, followed by a detailed discussion on the material selection and detection mechanisms of the devices. The review also provides an assessment of the application achievements of μPADs in medical diagnostics, environmental analysis, and food safety monitoring. Finally, current challenges in the field are summarized and future research directions and prospects are proposed.

## 1. Introduction

Diseases and residual food additives have been seriously endangering human health for a long time. In addition, heavy metal ions and organic pollutants significantly impact the ecological environment due to the discharge of industrial wastewater. Therefore, developing efficient detection techniques is critical for addressing challenges in multiple fields. Surface-enhanced Raman spectroscopy (SERS) [1], immunoassays (IAs) [2], DNA microarray method [3] and magnetic resonance imaging [4] are used for the detection of biochemical indices in the human body. Atomic absorption spectrometry [5], the electrochemical method [6], inductively coupled plasma mass spectrometry (ICP MS) [7], and high-performance liquid chromatography (HPLC) [8] are used to determine heavy metal ions or toxins in the environment. Enzyme-linked immunosorbent assay (ELISA) [9], laser-induced fluorescence spectrometry [10], and mass spectrometry (MS) [11] are used for the determination of residual drugs and microorganisms in food. Although these techniques can detect biomarkers, they still have drawbacks such as complicated sample preparation, expensive equipment, and a complicated detection process.

**Figure 1:**
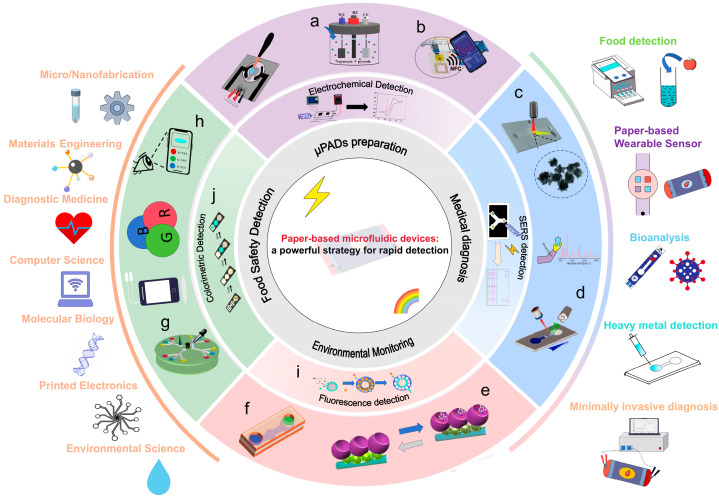
A schematic of μPADs and their applications in various fields. (**a**) Electrochemically assisted sensor. Reproduced from Ref. [29], with permission from Elsevier, 2024. (**b**) Smartphone-assisted sensor. Reproduced from Ref. [30], with permission from Elsevier, 2023. (**c**) Transparent nanopaper-based devices. Reproduced from Ref. [31], with permission from Marketplace, 2020. (**d**) SERS-based assisted sensor. Reproduced from Ref. [32], with permission from Elsevier, 2022. (**e**) Molecularly imprinted μPADs. Reproduced from Ref. [33], with permission from ACS, 2018. (**f**) Optofluidic μPADs. Reproduced from Ref. [34], with permission from Springer Nature, 2025. (**g**) Carbon quantum dots μPADs. Reproduced from Ref. [35], with permission from Springer Nature, 2023. (**h**) Colorimetric-assisted sensor. Reproduced from Ref. [36], with permission from Elsevier, 2024. (**i**) Fluorometric μPADs. Reproduced from Ref. [37], with permission from Springer Nature, 2021. (**j**) Colorimetric Method. Reproduced from Ref. [38], with permission from Elsevier, 2024.

Microfluidic paper-based analytical devices (μPADs) are considered an ideal platform for biomarker detection due to their versatile structures, flexible operational capabilities, and compatibility with various detection technologies [12]. Different materials are used to prepare microfluidic devices. Glass [13] and silicon [14] are early microfluidic device materials, and their limitations include high stiffness, impermeability, and high cost. In addition, the porosity and biocompatibility of hydrogels mean they are mainly used in bio-microfluidic devices [15]. However, their application in complex physiological environments poses a challenge, as they are highly susceptible to humidity, temperature, and mechanical stress, which can lead to reduced adhesive strength. Recently, paper-based materials have been recognized as a highly promising substrate for microfluidic devices, offering numerous advantages such as being pollution-free, highly flexible, low-cost, and featuring well-established fabrication processes [16]. Paper-based materials also boast wide raw material availability, processability, printability, and a large specific surface area. Notably, capillary action of paper eliminates the need for external pumping mechanisms when constructing microfluidic devices [17]. Consequently, μPADs serve as versatile biochemical analysis platforms offering operational simplicity and cost-effectiveness, making them ideal for point-of-care testing (POCT) [18]. Typical substrates for μPADs include widely used filter paper [19], nitrocellulose (NC) paper [20], and cellulose nanofiber paper (CNP) [21]. Yang et al. developed a paper-based biosensing platform through functional modification of qualitative filter paper, enabling plasma and erythrocyte separation from whole blood samples [22]. This platform achieved high-sensitivity synchronous detection of uric acid, creatinine (CR), and albumin (Alb), with detection limits of 0.1127 mM, 0.2978 μM, and 0.7696 mg/mL, respectively. Lin et al. fabricated a μPAD platform utilizing polyurethane acrylate-patterned NC membranes for dual detection of alpha-fetoprotein and carcinoembryonic antigen (CEA), attaining detection limits as low as 136 pg/mL and 174 pg/mL, which surpass the sensitivity required for clinical diagnostics [23]. Additionally, Zong et al. designed a transparent paper-based sensing platform that harnesses wavelength-dependent absorbance and transmittance for multi-analyte detection. This platform successfully detected both bovine serum Alb and cholesterol, achieving detection limits of 0.1 μM and 0.1 mM, respectively [24]. With the combination of various functional materials with paper fibers, this study involved the acquisition of sensing capabilities. As a result, more and more advanced analytical methods can also be realized on μPADs. Paper-based materials have become a key supporting material for the development of POCT, and their diverse material selection and functionalization strategies provide a broad application prospect for rapid detection [25,26,27,28]. Figure 1 illustrates the common detection principles and related research applications of μPADs. These platforms enable diverse applications spanning medical diagnostics, food safety testing, and environmental safety testing.

While significant advances have been made in μPADs, their potential to replace traditional, mature rapid analysis methods remains difficult, and the reliability of their practical applications requires further validation. This paper aims to systematically review μPADs developed in recent years for medical diagnostics, environmental monitoring, and food testing. This paper focuses on analyzing their key characteristics, including manufacturing processes and detection methods, while providing detailed information on commonly used paper-based materials. Finally, current challenges and future research prospects are outlined to facilitate the real-world implementation of μPAD-based technologies.

## 2. Key Components and Technologies of μPADs

### 2.1. Core Substrate Materials for μPADs

With the continuous advancement of materials science and manufacturing technolgy, the variety of paper-based materials available for fabricating microfluidic devices has expanded significantly [39,40,41]. In addition to traditional cellulose paper, materials such as glass fiber [42], cotton fiber [43], NC [43,44], cellulose acetate (CA) [45] and various cellulose-based composites [46] are now widely used.

Traditionally, paper-based materials were defined as porous materials composed of cellulose [47,48]. However, with the expansion of material systems and growing functional requirements, the concept has expanded to include various porous, flexible, and hydrophilic membrane materials [49]. Based on chemical composition, paper-based materials can be categorized into two major classes: cellulose-based [50] and non-cellulose-based materials [51], forming the material foundation of μPADs. Table 1 presents the common substrate materials, key properties, and how these materials are processed for μPADs.

#### 2.1.1. Cellulose-Based Substrate Materials

Various cellulose-based paper materials have been widely used in microfluidic analysis. Cellulose is a linear polymer [80], composed of D-glucopyranose units linked by β-(1–4) glycosidic bonds [81]. The abundance of hydroxyl groups (-OH) on its molecular chains confers hydrophilicity and biocompatibility, beneficial for subsequent chemical modification [82,83].

The advantages of cellulose paper as a substrate include low consumption of samples and reagents, good biocompatibility, portability, disposability, and flexible structural design [84]. The key parameters of cellulose paper encompass basis weight, thickness, porosity, particle retention capacity, filtration rate, capillary adsorption rate, and whiteness, which all influence its fluid behavior and analytical performance [85]. Among these, paper thickness significantly affects fluid transport and detection performance. For example, thinner paper offers lower flow resistance and higher solution transport rates, which can shorten reaction times, such as color development, and consequently, accelerate device response and readout speed [86]. Additionally, thickness determines the dimensions of hydrophilic channels confined by hydrophobic barriers, further influencing optical path length, light scattering effects, analytical sensitivity, and required liquid volume. Porosity refers to the percentage of pore volume in a material relative to its total volume. In paper-based materials, it refers to the proportion of the tiny channels and spaces formed between fibers. The high porosity of μPADs usually means that the resistance of fluid flow is smaller, the capillary force is stronger, and the flow velocity of the liquid sample is faster. This shortens the transmission time of the sample from the sample addition area to the detection area, enabling rapid diagnosis. Common materials used for cellulose-based microfluidic devices include filter paper, NC membranes, CA membranes, and CNP.

Filter paper is the most used base material for manufacturing μPADs, composed primarily of cellulose derived from wood or cotton pulp [52] and is manufactured using traditional papermaking processes, including pulping, sheet formation, and drying, offering significant advantages, such as low cost and suitability for large-scale production. However, constructing microfluidic devices often requires additional patterning techniques, including wax printing [87], photolithography [88], inkjet printing [89], or laser cutting [90], to form hydrophobic barriers. Regarding physical properties, filter paper exhibits excellent hydrophilicity and capillary action, enabling autonomous fluid transport. Filter paper, with its inherent porosity and large surface area, facilitates efficient reactant loading and molecular capture. Nevertheless, the material’s disordered fiber arrangement and broad pore size distribution have limitations, including poor fluid control precision, low reproducibility, and limited mechanical strength. Consequently, filter paper-based μPADs are generally not suitable for high-sensitivity diagnostic applications and are mainly used for educational demonstrations [91], preliminary environmental screening [92], and basic food nutrient monitoring [53].

For NC, each glucose unit on the cellulose macromolecular chain has three hydroxyl groups that can undergo esterification reactions with acids, among which the product formed with nitric acid. NC membranes can be prepared using methods such as phase inversion [54], spin coating [93], or electrospinning [94], with raw materials derived from various sources such as Miscanthus fiber [95], bacterial cellulose [96], and esparto grass [97]. Another important characteristic of the NC membrane is its ability to effectively combine proteins through hydrophobic interactions and hydrogen bonds [98,99,100]. In lateral flow immunoassays (LFIAs), this protein binding ability plays a crucial role in the fixation of antibodies and antigens, enabling specific molecular interactions to occur smoothly in specific regions on the membrane, namely the test line and control line. Consequently, NC membranes serve as the core material in LFIAs technology [101,102,103,104,105], widely used in pregnancy tests, rapid infectious disease screening, cancer biomarker detection [56], and food safety applications [106]. However, generating a robust hydrophobic structure within the NC membrane to fabricate μPADs remains a significant challenge [107]. This requires further enhancement of its performance through material modification. Tang et al. introduced chitosan into NC membranes, which reduced pore size, increased porosity, and enhanced bio-immobilization capacity, resulting in a tenfold enhancement in the analytical sensitivity for hepatitis B virus [108].

CA membranes are produced through the acetylation of cellulose, offering good biocompatibility and tunable electroosmotic flow properties [109,110]. CA membranes can be formed into structures, such as flat sheets, hollow fibers, and nanofiber membranes, via phase inversion processes [111,112,113,114]. The residual hydroxyl groups on the anhydro glucose units facilitate modifications such as oxidation, etherification, esterification, grafting, and cross-linking, expanding the material’s functionality [57,115,116,117,118,119,120]. In microfluidic applications, CA membranes are commonly used for electrophoretic separation, filtration, and the construction of electrochemical biosensors [121,122,123,124]. In protein separation applications, their performance surpasses NC membranes [125,126,127]. The material features an interconnected three-dimensional (3D) porous structure that enhances the immobilization and capture of biomolecules [128], making it suitable for detecting analytes such as organic pollutants, bacteria, and cancer biomarkers [58]. However, CA membranes face a fundamental challenge in paper-based microfluidics: weak biomolecular binding capacity. Their surface chemistry results in much lower physical protein adsorption than NC membranes. This characteristic implies that it is often necessary to activate it (for example, by using its residual hydroxyl groups for chemical cross-linking) or to develop new immobilization strategies [129,130].

CNP is a nanocellulose film made by β-1,4-glycosidically bonded glucose units [131,132]. Mechanical or chemical treatment at the nanoscale achieves fine alignment and densification of fibers [59,133]. Common preparation methods include film formation using cellulose nanofibrils [134] or impregnating conventional filter paper with resins such as polymethyl methacrylate (PMMA) to eliminate light scattering and achieve high optical transparency [31,135,136]. CNP exhibits an ultra-smooth surface, which is often rough below 25 nm, along with uniform nanopores, high flexibility, and biodegradability [60,137]. The high optical transmittance significantly reduces background noise and improves the signal-to-noise ratio and sensitivity in fluorescence and chemiluminescence detection, overcoming the opacity issues of traditional filter paper. CNP suits μPADs requiring high-precision optical quantitative analysis and shows broad prospects in environmental monitoring, biosensing, medical diagnostics, and food safety [138,139]. For example, Sangkaew et al. utilized its efficient immobilization of glucose oxidase to achieve instrument-free visual quantitative glucose detection, demonstrating excellent sensitivity and accuracy in biomedical applications [140]. However, the uniform but tiny nanopores of CNP not only facilitate the formation of a smooth surface, but are also prone to being blocked by biological macromolecules or particulate matter in the sample, thereby affecting the smooth flow of the liquid, particularly when dealing with complex real samples, including blood and sewage.

#### 2.1.2. Non-Cellulose-Based Substrate Materials

While non-cellulose-based materials do not originate from natural cellulose, their porous membrane morphology and unique functional properties have demonstrated significant potential for specialized applications in μPADs [141]. Common non-cellulose paper-based materials include polyethersulfone (PES), glass fibers, PMMA, polydimethylsiloxane (PDMS), and composite materials.

PES membranes are typically fabricated from PES polymers via phase inversion processes [61]. Additionally, PES can be processed into microfluidic devices with complex structures through hot pressing or injection molding. Regarding physicochemical properties, PES membranes exhibit high mechanical strength, low protein adsorption, and desirable porosity [62]. The porous structure of PES supports efficient retention and diffusion of reagents within the membrane, while its low protein adsorption minimizes non-specific binding of enzymes and nucleic acids. These properties collectively enable effective isothermal nucleic acid amplification within the pores [142]. Compared to cellulose paper, PES maintains efficient liquid diffusivity and reaction kinetics even when stacked in multiple layers, thereby preserving high sensitivity. PES membranes are primarily used in biosensing and microfluidic analysis, particularly in integrated detection systems. For instance, in food safety monitoring, PES membranes can serve as integrated platforms for nucleic acid extraction, recombinase polymerase amplification (RPA), and LFIAs, enabling rapid and highly sensitive detection of *Salmonella enterica* [143]. However, the unmodified PES is essentially hydrophobic. In μPADs driven by capillary forces, the hydrophobic nature would prevent the spontaneous infiltration and flow of aqueous solutions. To make it applicable to microfluidics for aqueous samples, the surface of PES must be hydrophilized. For example, the incorporation of modified microcrystalline cellulose at varying concentrations into PES membranes enhanced their affinity and adsorption capacity for heavy metal ions. These modifications also improve membrane hydrophilicity and porosity, increasing filtration flux and antifouling performance while maintaining high mechanical strength [144]. In biomedical applications, PES membranes modified with reduced graphene oxide can be used to construct biomimetic self-driven microfluidic devices, for efficient clearance of urea in blood, mimicking glomerular filtration function [63].

Glass fiber paper is primarily composed of inorganic glass fibers. Its chemical structure is based on silica tetrahedra as the fundamental units, which form a continuous 3D network connected by bridging oxygen atoms [70]. Metal oxides such as sodium, calcium, and boron are incorporated to modulate its physicochemical properties. The material is typically manufactured via a wet-laid papermaking process, which involves dispersing glass fibers in water, followed by sheet formation, dewatering, and drying. It can also be impregnated with polymers and hot-pressed to form composite-reinforced materials [145]. Glass fiber paper exhibits high mechanical strength, excellent thermal stability that can withstand temperatures over 500 °C, a low coefficient of thermal expansion, resistance to chemical corrosion, and tunable hydrophilicity/hydrophobicity [145]. Furthermore, it is biochemically inert [146]. However, glass fiber paper lacks abundant chemically modifiable active functional groups, such as hydroxyl groups found in cellulose. Therefore, specific and stable functionalization modification is required. After surface modification, Fang et al. employed GO-modified glass fiber paper coupled with fluorophore-labeled single-stranded DNA to achieve rapid and low-cost detection of biomacromolecules [147]. Axel et al. deposited nanoparticles (NPs) onto glass fiber paper-based SERS substrates via spray deposition, showing a clear enhancement in SERS performance relative to cellulose paper [148]. In μPADs, glass fiber paper is a high-performance porous substrate for constructing microchannels, reaction zones, or separation units. Upon surface functionalization through methods such as hydrophobic modification, the glass fiber paper can be used for oil–water separation, extraction, and phase separation. Moreover, its robust mechanical strength and thermal stability support long-term use under diverse conditions [149].

Polymer materials have gained increasing attention in microfluidic manufacturing due to their relatively low cost, good biocompatibility, and high chemical resistance. Commonly used polymer materials include thermoplastic plastics, such as PMMA, and elastomers such as PDMS [150]. PMMA is widely used due to its low material cost, sound thermal processing, and optical properties [64,65,66]. However, the hydrophobic nature of PMMA will hinder the fluid transmission driven by capillary action, requiring additional hydrophilic modification or pump-driven liquid delivery. The paper mixture platform already has many rapid diagnostic applications based on various detection technologies. Therefore, PMMA/paper hybrid μPADs are developed. The capillary action of the paper can achieve pump-free self-driven operation, thereby reducing the complexity and cost of the equipment [151]. For example, Lei et al. developed a 3D microfluidic platform based on paper and PMMA for cell culture, which is used to study cell interactions [67]. Taheri et al. used a PMMA/paper hybrid microfluidic chip to simultaneously determine the amino acids arginine and valine in plasma [152].

PDMS has become the most popular polymer in the research of μPADs due to its ease of manufacture, transparency, low electrical conductivity, and elasticity [68]. Compared with common silicon-based materials, PDMS is cheaper and easier to cure on the template at an appropriate temperature. However, PDMS materials have a high hydrophobicity, making it difficult for water-based solutions to flow through the microchannels, resulting in bubbles and non-specific adsorption of biological molecules [153]. Long et al. used O_2_ plasma treatment combined with polyethylene glycol (PEG) coating to modify the surface of PDMS materials, achieving a long-term stable improvement in their hydrophilicity [154]. Lin et al. aimed to enhance the hydrophilicity of PDMS μPADs [69] by grafting a series of polyamidoamine (PAMAM) dendrimer polymers onto the PDMS, and then silanizing with γ-epoxypropoxypropyl triethoxysilane. Compared with the unmodified PDMS, its contact angle decreased from 108.1° to 31.8°. PDMS paper-based microfluidics combines the precise fluid control capability of PDMS with the low cost and capillary-driven characteristics of paper-based materials to construct functionally enhanced composite chips [155,156]. This technology employs paper as a substrate for reaction and detection, enabling reagent storage and sample transport, while a PDMS layer provides encapsulation, evaporation control, and functional integration, thereby boosting chip stability and detection sensitivity [157,158,159]. It is widely applied in POCT, environmental monitoring, and other fields [160]. However, the interface bonding strength and material compatibility remain key technical challenges that must be overcome [161].

Composite paper is structured primarily with natural cellulose fibers as the matrix, integrated with functional nano- or micro-scale materials. The diverse processing methods include slurry blending and precipitation, electrodeposition, printing, and impregnation [77]. Composite paper exhibits a range of favorable physicochemical properties, such as high specific surface area and well-developed porosity [76]. These characteristics facilitate fluid transport and enhance reaction efficiency, demonstrate strong catalytic activity and chemical stability, and retain the flexibility and processability inherent to paper-based materials. In applications, composite paper is often employed within μPADs as reaction zones, detection areas, or modified electrode layers, enabling highly sensitive detection of biomarkers and environmental pollutants [43]. For instance, Mahdee et al. developed a cost-effective passive microfluidic paper-plastic hybrid device for blood typing. This device enabled RBC agglutination without saline washing, allowing agglutination/nonagglutination classification via the naked eye and mean intensity images [72]. Similarly, Mohamad et al. grafted polymeric ionic liquids onto commercial filter paper via impregnation to create a novel μPADs for thin-film microextraction of multiple antibiotics in environmental water samples [73]. Thanks to its integrated design, such materials support pump-free fluid delivery, in situ chemical reactions, and signal output functions. As a result, composite μPADs are widely used in POCT across diverse fields, including environmental monitoring, disease surveillance, and food safety, significantly enhancing the analytical performance and practical utility of microfluidic devices [162,163,164].

### 2.2. The Fabrication of μPADs

μPADs integrate sample preparation, reaction, separation, and detection, and achieve operation miniaturization [165]. Within a μPAD, the liquid flows by capillary action of the microchannels or the paper, ensuring that the entire experimental analysis can be carried out smoothly [166]. Therefore, the paper used to prepare μPADs should have good wetting properties, mechanical properties, and stability. To translate these material properties into functional devices, the fabrication process typically involves three key stages: substrate selection and pretreatment [166], creation of microfluidic channels [167], and final device encapsulation and integration [165].

#### 2.2.1. Substrate Selection and Pretreatment

The selection and pretreatment of paper substrates represent the initial step in fabrication. Substrate selection follows several core principles [26]: (i) capillary drive capability of the substrate; (ii) microchannel patterning; (iii) favorable modifiability for subsequent loading of biomolecules. Traditional microfluidic chips often rely on external pressure to drive fluid flow, whereas paper substrates offer the unique advantage of spontaneous capillary-driven transport [168]. This capability stems from the intrinsic structure of plant fiber paper: its cell walls form a porous network of lignocellulose, creating a highly permeable matrix with high porosity. This geometric structure, combined with cellulose’s inherent hydrophilicity, enables μPADs to achieve spontaneous liquid transport without external power [169]. Therefore, substrate selection requires a comprehensive evaluation of capillary flow properties, primarily including porosity, chemical compatibility, mechanical strength, and cost. Pretreatment involves cleaning, flattening, or surface energy modification to prepare for subsequent patterning processes [170].

#### 2.2.2. Patterning of Microfluidic Channels

**Figure 2:**
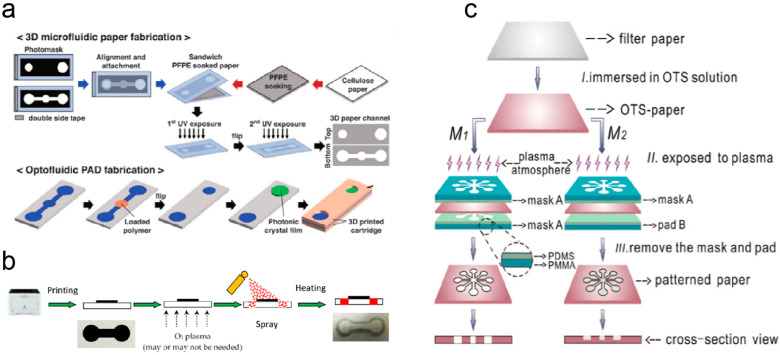
Fabrication of μPADs. (**a**) Photolithographic processing of μPADs. Reproduced from Ref. [34], with permission from Springer Nature, 2025. (**b**) Printing processing of μPADs. Reproduced from Ref. [183], with permission from MDPI, 2022. (**c**) Plasma etching processing of μPADs. Reproduced from Ref. [38], with permission from Elsevier, 2024.

The core step is constructing hydrophilic/hydrophobic channels to guide the directional flow of the sample, involving patterning the paper to define precise fluidic paths. The processing of μPADs involves a variety of methods (Figure 2). Commonly used techniques are printing [171,172], plasma etching [173,174], and photolithography [175]. Initially, μPADs were processed by constructing hydrophobic regions on the surface of cellulose paper by UV photolithography [175]. Using UV light to crosslink the photoresist copies the mask pattern onto it, which is then transferred to the paper substrate by etching. Apart from photoresist, wax is the most common hydrophobic material. Paraffin wax is pre-attached to the paper substrate in a defined pattern, melts when heated, and penetrates the paper substrate, eventually forming a hydrophobic barrier [176,177]. Batik eliminates the need for chemicals in photolithography and is a much simpler process. Among printing methodologies such as inkjet printing [178], screen printing [179], and flexographic printing [180], hydrophobic inks are printed on a paper substrate to create liquid channels. Nozzles in an inkjet printing device extrude ink and spray a mist of ink droplets onto the surface of the paper substrate, where the droplets draw a predetermined pattern [181]. Hydrophobic inks achieve higher-resolution channels through inkjet printing without the need for heat, as with wax. Another method is plasma etching, where treated paper is selectively etched to establish hydrophilic/hydrophobic patterns [182]. Due to high efficiency and low cost, plasma etching is considered one of the viable techniques for processing paper substrates.

#### 2.2.3. Device Encapsulation and Integration

Encapsulation and integration are critical for transforming patterned paper into functional devices [160]. This stage bonds transparent films to paper substrates through hot pressing, adhesive bonding, or lamination processes, forming sealed chambers that prevent fluid leakage and external contamination. Encapsulation also enhances mechanical stability, defines sample introduction ports and detection windows, and controls sequential fluid flow in multilayer designs [166]. The encapsulated paper-based chips are then cut to the desired dimensions and shapes, followed by drilling, polishing, and trimming.

To achieve these design functions, the key lies in precisely controlling the flow of liquid within the device. When manufacturing paper-based microfluidic devices, one key point is the flow of the liquid, which is controlled by factors such as permeability and geometry [184]. This equation is derived from Jurin’s law [185] and the Hagen–Poiseuille equation [186]. The average velocity of fluid flow can be summarized as [187]:

(1) $$\nu =\frac{\gamma cos\;\theta }{4\mu }\frac{1}{L},$$

where ν represents the average flow velocity, γ is the surface tension, θ is the contact angle, μ is the viscosity, and is the distance traveled by the liquid.

Once the medium is fully wetted, and the subsequent flow is laminar. For such flow conditions, the macroscopic behavior follows Darcy’s law, which governs the linear dependence of fluid flow velocity on the pressure gradient in a porous medium [188]. This relationship [189,190,191,192] is generally expressed as follows:

(2) $$\nu =-\frac{K}{\mu }\nabla P,$$

where ν represents the average flow velocity, K denotes the medium’s permeability, μ is the dynamic viscosity, and ∇P signifies the pressure gradient.

## 3. Applications of μPADs

### 3.1. Detection Methods for μPADs

**Figure 3:**
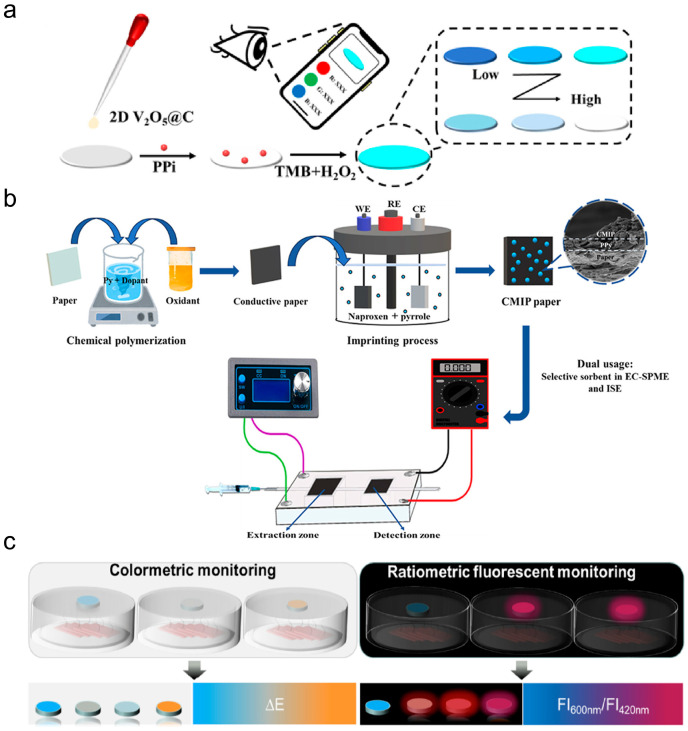
Detection methods for μPADs. (**a**) Colorimetric pyrophosphate (PPi) detection. Reproduced from Ref. [36], with permission from Elsevier, 2024. (**b**) Electrochemical naproxen sensing. Reproduced from Ref. [29], with permission from Elsevier, 2024. (**c**) Fluorescent biogenic amine monitoring. Reproduced from Ref. [196], with permission from Elsevier, 2022.

μPADs are often modified to enable them to detect and analyze markers. Various analytical methods have been implemented in μPADs, such as colorimetric methods [193], electrochemical methods [194], and fluorescence methods [195]. Figure 3 demonstrates the analytical approach of the μPADs. Combining paper-based and analytical techniques makes μPADs simultaneously fast, miniature, and convenient for practical applications.

#### 3.1.1. Colorimetric Detection

Colorimetric methods determine analytes through color development reactions, offering high intuitiveness and universality. The white background of paper substrates provides excellent contrast, while their inherent wettability passively drives analytes toward the detection zone [197,198]. Therefore, colorimetric methods do not require sophisticated instrumentation to obtain visual results from the color change in the test area before and after the reaction. Common colorimetric reactions are pH-induced chromatography [199], enzymatic reactions [200], and complexation reactions [201]. Moreover, pH-induced chromatography is based on the principle that pH-sensitive materials change color in response to a change in the acidity or alkalinity of their surroundings, since pH can be an important indicator of environmental and biological significance, such as water quality [202], blood [203], and sweat [204]. Colorimetric analysis based on pH-induced color change is widely used in the ecological environment [205] and human health testing [206]. In addition, most chemical reactions in living organisms are enzymatic. Chromogenic substrates react with enzymes to produce colored products that can be detected using absorbance enzyme markers [207]. Colorimetric analyses based on enzymatic reactions can be used for the detection of diverse forms of analytes, such as small molecules [208], cells [209], and microorganisms [210]. Metal ions in the environment are often analyzed colorimetrically using complexation reactions [211]. A ligand binds to the metal ion to form a stable coordination complex. The color of the central atom changes due to the change in electron energy before and after complexation [212].

#### 3.1.2. Electrochemical Detection

Electrochemical methods measure changes in electrical potential through electrodes on a paper base [213], which can detect many electrochemically active analytes such as small molecules [214], proteins [215], and metals [216]. Currently, methods for manufacturing electrodes on paper substrates have been significantly developed, such as inkjet printing [217], screen printing [218], sputtering [219], and laser scribing [220]. Carbon electrodes are attractive because of their affordability and high conductivity [221]. In contrast, precious metal electrodes have superior electrochemical activity [222]. It has been found that modifying carbon electrodes with graphene [223] or Cu/Co double-doped CeO_2_ nanospheres [224] can improve the electrochemical performance of μPADs. In recent years, significant progress has been made in related research. For example, Mei et al. developed a wearable 3D electrochemical μPAD for real-time monitoring of specific sweat constituents, including glucose, lactate, and Cl^−^ [225]. Fabricated via a layer-by-layer approach, this device integrates a 3D paper-based microfluidic network with an integrated electrochemical sensor. The modification with AuNPs and Prussian blue enhances both conductivity and detection sensitivity. Yang et al. developed an electrochemical μPAD utilizing MBene and AuNPs for the detection of circulating tumor DNA (ctDNA) in mouse serum [226]. MBene provides a highly conductive electrode interface with a large specific surface area, while AuNPs further enhance conductivity and offer high-density, low-impedance sites for immobilizing DNA probes, enabling sensitive ctDNA detection.

#### 3.1.3. Fluorometric Detection

Fluorescent μPADs are powerful tools for the detection of analytes such as organic compounds [35], heavy metals [227], pathogens [228], and biomolecules [147] due to their high sensitivity. Fluorophores absorb energy at specific wavelengths and re-emit it at other wavelengths, resulting in visible fluorescence [229]. Various types of fluorescent probes have been used in fluorescent μPADs, such as fluorescent dyes [230], aggregation-induced emission (AIE) materials [231], nanoclusters [232], and quantum dots [232]. Recently, Jiang et al. developed a coumarin-derived biomass-based fluorescent material for a bifunctional paper-based sensing platform [233]. This platform is designed for determining Cu^2+^ concentration in drinking water and monitoring meat freshness by recording real-time color changes in the paper-based sensitive material during its interaction with Cu^2+^ and H_2_S. Then, by integrating the fluorescent sensing platform with a smartphone application that reads RGB values, the system achieves non-destructive, intuitive, and direct monitoring of Cu^2+^ levels and raw meat freshness. Li et al. developed a dual-probe colorimetric-fluorescent paper-based sensor with nitrogen-doped carbon dots, copper nanoclusters, and AuNPs for urinary CR detection [234]. The sensing mechanism relies on urinary CR binding that disrupts the phenomenon of fluorescence resonance energy transfer (FRET) occurring in nitrogen-doped carbon dots and AuNPs, thereby restoring green fluorescence while quenching the red emission from copper nanoclusters. Furthermore, the combination of paper-based sensing and smartphone technology allows the sensor to deliver highly sensitive and selective rapid detection across the 1–50 mM concentration range [234]. Yin et al. fabricated a wearable paper-based microfluidic patch functionalized with red–green dual-emission fluorescent probes based on CdTe quantum dots coordinated with Tb^3+^ and Eu^3+^ [235]. This fluorescence platform was enhanced 45-fold by an aggregation-regulated antenna effect to quantitatively detect four fluoroquinolone antibiotics and distinguish nine commercial eyedrop solutions within about 5 min.

### 3.2. Medical Diagnosis

With the aggravation of environmental pollution and frequent outbreaks of viruses, medical diagnosis shows great value [38]. Since there is uncertainty in the occurrence and development of diseases, accurate and timely detection plays a significant role in preventing and treating diseases [160]. In recent years, μPADs have been used to rapidly detect human blood, tumor biomarkers, and infectious microorganisms.

#### 3.2.1. Blood

Human blood consists of complex components, including plasma and blood cells rich in proteins, electrolytes, organic compounds, erythrocytes, leukocytes, and platelets [236]. Blood is regarded as an essential indicator for evaluating the health of an individual and is widely used to detect various diseases such as Alzheimer’s disease [237], lung cancer [238], and pancreatic cancer [239].

**Figure 4:**
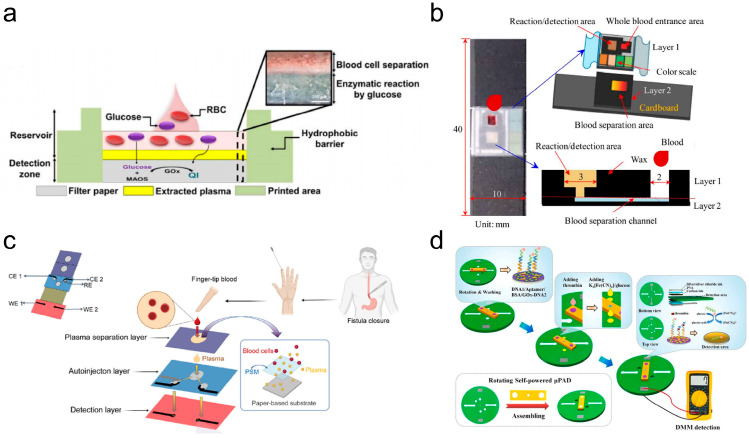
μPADs for blood testing. μPADs for point-of-care blood testing: designs and functions. (**a**) Separation of blood cells and glucose detection by the 3D-μPAD. Reproduced from Ref. [243], with permission from ACS, 2019. (**b**) Major components of the proposed 3D μPAD. Reproduced from Ref. [244], with permission from Elsevier, 2018. (**c**) Multi-functional paper-based device’s operation procedures and basic functions. Reproduced from Ref. [245], with permission from Elsevier, 2022. (**d**) The detection process of a self-powered μPAD. Reproduced from Ref. [249], with permission from Elsevier, 2022.

Currently, venipuncture and finger-prick are standard methods of blood sampling. Vein puncture testing blood with laboratory instruments can yield accurate test results. However, waiting for the results often takes a long time. The rapidity of blood testing is achieved by collecting capillary blood at the fingertip and analyzing the sample with a POC blood analyzer [240]. However, successive drops of blood may cause compositional changes that can affect the accuracy of the test results. In contrast, μPADs that enable rapid diagnostics are powerful tools for testing blood. Although whole blood samples are the easiest to extract, red blood cells interfere with the analyte signal in colorimetric and optical analyses [241]. Plasma in conventional assays needs to be separated from whole blood with the help of a centrifuge and precipitated into μPADs [242]. Later, μPADs were combined with filters such as plasma separation membranes (PSMs) to integrate the sample preparation steps. Park et al. fabricated a PSM-integrated 3D μPAD (Figure 4a) [243]. PSMs were superimposed on a paper substrate, and then a digital light processing printer was used to create the detection area and the storage library, eliminating the need for additional assemblies. As shown in Figure 4b [244], Fu et al. proposed a 3D μPAD device for quick and easy detection of whole blood ALB [244]. Whole blood samples diffuse by capillary action through a separation channel into a reaction zone, where ALB binds to bromocresol green dye. The resulting absorbance change is transmitted via a smart interface to a mobile phone. Capillary action in paper-based material also provides the energy required for plasma separation. For example, Cai et al. prepared an origami μPAD enabling effective blood cell segregation and simultaneously detecting C-reactive protein and prealbumin (Figure 4c) [245]. The device integrated three major functions of plasma isolation, self-driven sample introduction, and selective target identification, and could accurately quantify gastrointestinal fistula biomarkers in whole blood samples. LFIAs, based on specific antibody–antigen recognition, have the advantages of sensitivity, rapidity, and accuracy [246]. Chen et al. developed an approach for analyzing human chorionic gonadotropin in serum by integrating electrochemical immunofiltration analysis into μPADs fabricated via photolithography and screen-printing [247]. To address the inadequate hydroxyl groups on pure cellulose paper for securing antibodies, the researchers covalently anchored the antibody by converting hydroxyl groups to aldehyde groups via periodate oxidation. Due to the absence of a support pump or pressure source in the base μPADs, liquid flow relies entirely on the paper’s well-defined hydrophilic and hydrophobic regions [248]. Although promising results have been achieved, research to enhance the control of fluids continues. Li et al. constructed a self-powered rotating μPAD using rotary valve technology for thrombin detection (Figure 4d) [249]. The integrated removable valve has flexible fluid control, allowing it to be easily manipulated for multiple operations. To precisely control the volume of the fluid, Fan et al. developed a digital μPAD integrating droplet manipulation and analysis to determine the concentration of lithium ions in blood [250]. The liquid volume is defined by the dimensions of the thin film electrodes and the distance between the electrode plates, obviating the need for complex microfluidic channels. The device showed good performance agreement with conventional methods, showing promise for μPADs for minimally invasive diagnostics.

#### 3.2.2. Tumor Markers

Tumor markers are present in the patient’s tissues, body fluids, and excretions and are active substances, such as proteins and nucleic acids, that reflect tumorigenesis and progression [163,251]. In cancerous conditions, the concentration of such active substances often differs from normal levels and can be detected by immunological, biological, and chemical methods [252]. Therefore, rapid detection of tumor markers is critical for the clinical management of tumors, encompassing early screening, precise diagnosis, and accurate prognosis.

**Figure 5:**
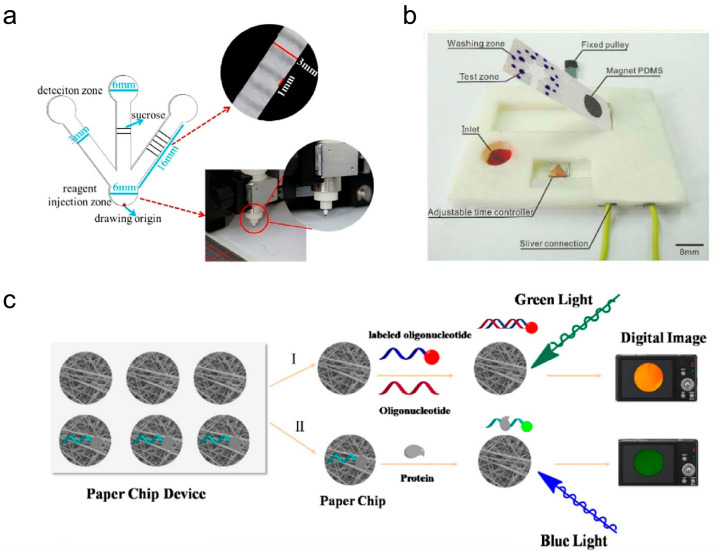
Integrated μPAD platforms for detecting tumor markers. (**a**) Schematic diagram of the μPAD based on CL and sugar drawing performances. Reproduced from Ref. [255], with permission from Elsevier, 2017. (**b**) A paper-based magnetic valve with tunable timing capability. Reproduced from Ref. [256], with permission from Elsevier, 2018. (**c**) Scheme of the paper-based FRET to detect proteins and nucleic acids. Reproduced from Ref. [147], with permission from Elsevier, 2016.

Many studies have used μPADs to detect protein-based tumor markers. Cai et al. prepared a label-free electrochemical immuno-μPAD to detect CEA using screen-printed electrodes [253]. Due to the formation of antibody–antigen immune complexes, the decrease in thionine reaction current was proportional to the concentration of the corresponding antigen. This strategy avoids labeling antigens and antibodies, making detecting CEA more efficient. In chemiluminescence (CL)-based μPADs, obtaining different migration times on the paper substrate is a feasible way to obtain the maximum CL signal required for quantitative analysis. Fu et al. added unequal amounts of sugar into the microchannels and established a soluble sugar barrier, generating different time-delay effects [254]. However, equal amounts of sugar were difficult to add again to the same position accurately, making the delay time’s reproducibility challenging. Liu et al. replaced the cutter of the process cutter with a pen filled with sugar solution [255]. Sucrose was plotted at the same position on the microchannel by setting the origin. The results showed that the delay time from one channel to another was about 20 s, and the maximum CL signal could be recorded in each detection zone (Figure 5a) [255]. Combining mechanical valves and programmed channels is an alternative approach to timed fluid control. The operational timing of the microvalve is directly dictated by the specific geometric parameters of the microfluidic channel. Therefore, it is essential to fabricate multiple timing channel designs to address the diverse requirements of valve driver systems. Feng et al. demonstrated a μPAD that integrated a magnetic valve and a tunable timer for chemiluminescence IA (CLIA) CEA [256]. The time was set via positional control of a conductive iron strip in the time controller. When the fluid contacts a conductive iron component, a circuit is formed, and the solenoid valve is activated, thereby initiating the CLIA reaction (Figure 5b) [256]. Serum carcinoembryonic antigen at different levels in clinical serum samples can be detected simultaneously within 16 min, together with high sensitivity and reproducibility.

Mutations in nucleic acids usually accompany tumorigenesis. Due to the low abundance of nucleic acids, detecting nucleic acid-based tumor biomarkers is difficult. Kong et al. constructed a μPAD using the FRET method [147]. GO was used as a bursting agent in contact with a glass fiber chip, which could distinguish between single-stranded DNA (ssDNA) and double-stranded DNA (Figure 5c) [147]. Yet, another work shows that when fluorescently labeled ssDNA was tightly bound to GO, the fluorescence was immediately burst [257]. However, upon addition of the complementary strand, the DNA probe is released from the GO, and fluorescence is restored. In addition to tumors, other factors in the body may affect the expression levels of nucleic acid-based tumor biomarkers, such as infection and inflammation. Thus, simultaneous detection of multiple nucleic acid-based tumor biomarkers can reduce the probability of erroneous positive diagnosis. To address these limitations, Hou et al. fabricated vertically aligned graphene walls (GWs) on carbon fiber paper (CFP) via plasma-assisted chemical vapor deposition [258]. Electrodepositing AuNPs onto CFP/GWs substrate yielded a 3D sensor with high surface area and conductivity, enabling the simultaneous detection of tumor exosomal microRNAs with detection limits as low as 23.1 aM for miRNA-21 and 33.4 aM for miRNA-155.

#### 3.2.3. Infectious Microorganisms

In recent years, infectious microorganisms such as bacteria and viruses have seriously threatened human health [259]. More than 400 known infectious microorganisms are found in the human respiratory system [260], digestive system [261], genitourinary system [262], and skin [263]. Due to the extremely rapid spread of such microorganisms, rapid detection is essential for preventing infectious diseases [264].

**Figure 6:**
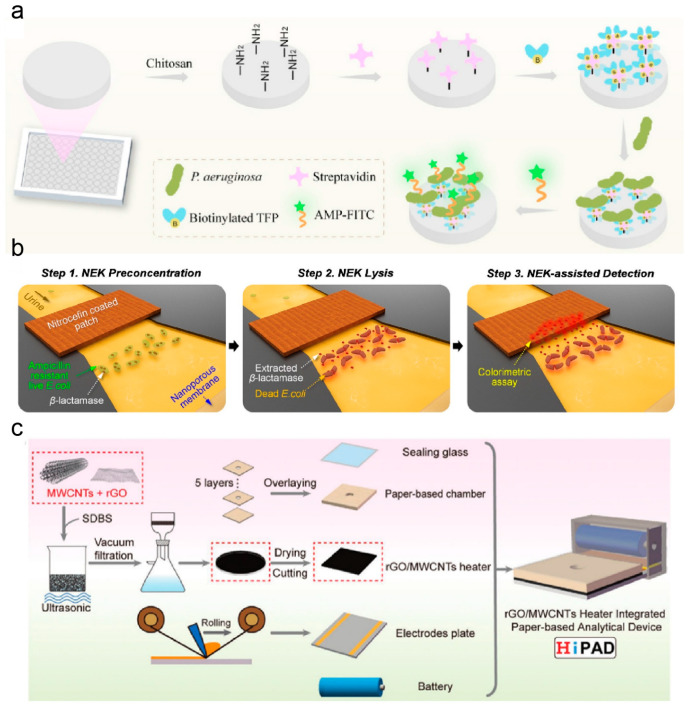
Advanced μPADs for POCT of infectious microorganisms. (**a**) The process for detecting Pseudomonas aeruginosa is based on a paper-based bacterial detection device. Reproduced from Ref. [228], with permission from Elsevier, 2023. (**b**) Fast electrokinetic-assisted detection of drug-resistant bacteria in urine. Reproduced from Ref. [269], with permission from Elsevier, 2022. (**c**) rGO/MWCNTs nano-circuit heater-assisted paper-based acid POCT. Reproduced from Ref. [271], with permission from WILEY, 2021.

Bacterial infections are common diseases caused by bacterial invasion and parasitism. Currently, pathogen culture remains the gold standard for diagnosing bacterial infections. Due to its time-consuming, costly, and complex instrumentation, developing convenient and timely assays is necessary. Lee et al. screened a variety of bacterial-systematic evolution of ligands by exponential enrichment of bacteria and integrated them into a μPAD system [258,265]. This new dual-aptamer μPAD possesses many advantages over its traditional-scale counterparts, such as faster detection times, higher specificity, and the capability to detect multiple pathogens simultaneously. The detection panel identifies *Acinetobacter baumannii*, *Escherichia coli*, and methicillin-resistant *Staphylococcus aureus* in under 35 min. Phage tail fiber protein (TFP) is an ideal bacterial capture molecule due to its non-cleaving activity and ability to recognize bacterial lipopolysaccharides, wall acids, and pore proteins. A paper-based bacterial detection device incorporating TFP was prepared by Fu et al. TFP derived from phage and an *Escherichia coli* expression system produced PaP1 and showed high sensitivity and selectivity for Pseudomonas aeruginosa (Figure 6a) [228]. Untreated bacterial infections can lead to pneumonia, meningitis, urinary tract infections, or bone and joint infections. Liu et al. designed a hybrid PDMS/paper μPAD based on a multiplexed colorimetric assay using paper-carrier cell culture arrays [266]. An integrated hydrophobic membrane valve allowed urine samples to be uniformly distributed into individual chambers, eliminating crosstalk between neighboring chambers, showing that the method completed multiple uropathogen identification and antimicrobial susceptibility testing assays within 15 h, which was significantly faster than the conventional method (3–4 days). The sensitivity still needs improvement as the urine sample mainly relies on the spontaneous lateral movement of a small volume of fluid to reach the detection window [267]. Hospital diagnostic and culture-based methods require nearly 2 days to complete testing [268], whereas Kim et al. reported an accurate nano-electrokinetic paper-based device that achieves the detection of common drug-resistant bacteria causing urinary tract infections in less than 7 min [269]. Once an external voltage was applied, the drug-resistant bacteria were preconcentrated near the nanopore membrane by equilibrium ion concentration polarization and radial swelling. The drug-resistant marker enzymes released via bacterial lysis then rapidly react with the chromogenic cephalosporin patch, causing a distinct colorimetric signal (Figure 6b) [269]. Viruses are smaller and more mutable than bacteria. Since the outbreak of novel coronaviruses, there has been a greater interest in the reliable detection of viruses [270]. Enabling rapid virus detection in resource-limited environments is essential for isolating patients and preventing the spread of viruses. Ding et al. fabricated a GO/multi-walled carbon nanotubes (MWCNTs) nano-circuit electrothermal heater by suction filtration. They integrated it into a μPAD to visualize SARS-CoV-2 by a colored Loop-mediated isothermal amplification reaction (Figure 6c) [271], achieving a broad dynamic range from 25 to 2.5 × 10^10^ copies/mL within 45 min. Jin et al. fabricated a μPAD platform that integrates a tension-spinning top for serum collection with a paper-based ELISA for the instrument-free quantification of SARS-CoV-2 receptor-binding domain-specific IgA/IgM/IgG antibodies [272]. Notably, the system also has the potential for early and late diagnosis of COVID-19 patients.

### 3.3. Environmental Monitoring

Now, the global ecological balance and ecological security are under serious threat, and environmental monitoring shows great value [273]. Due to the complexity and uncertainty of environmental changes, accurate and real-time monitoring of the environment is of significant importance for ecological protection, pollution prevention and control, and sustainable development [274]. In recent years, μPADs have been used to rapidly detect heavy metal ions and environmental pollutants [275].

#### 3.3.1. Heavy Metal Ions

With the rapid development of industry, the pollution of heavy metal ions in the environment has become a pressing problem [276]. Heavy metal ions are not biodegradable and can accumulate in the human body through food and drinking water [277,278]. Even at low concentrations, heavy metal ions can lead to serious toxic damage to the human body [279]. Meanwhile, heavy metals come in a wide variety, and their environmental impacts differ depending on their valence states. Therefore, developing methods to detect heavy metals with high sensitivity, specificity, and instant detection has always been a key concern in heavy metal pollution research [280]. Some new μPADs have been developed to efficiently detect heavy metal ions in the environment, such as Fe^2+^ and Ni^2+^ [281]. The recently reported methods for detecting heavy metals are shown in Table 2.

**Figure 7:**
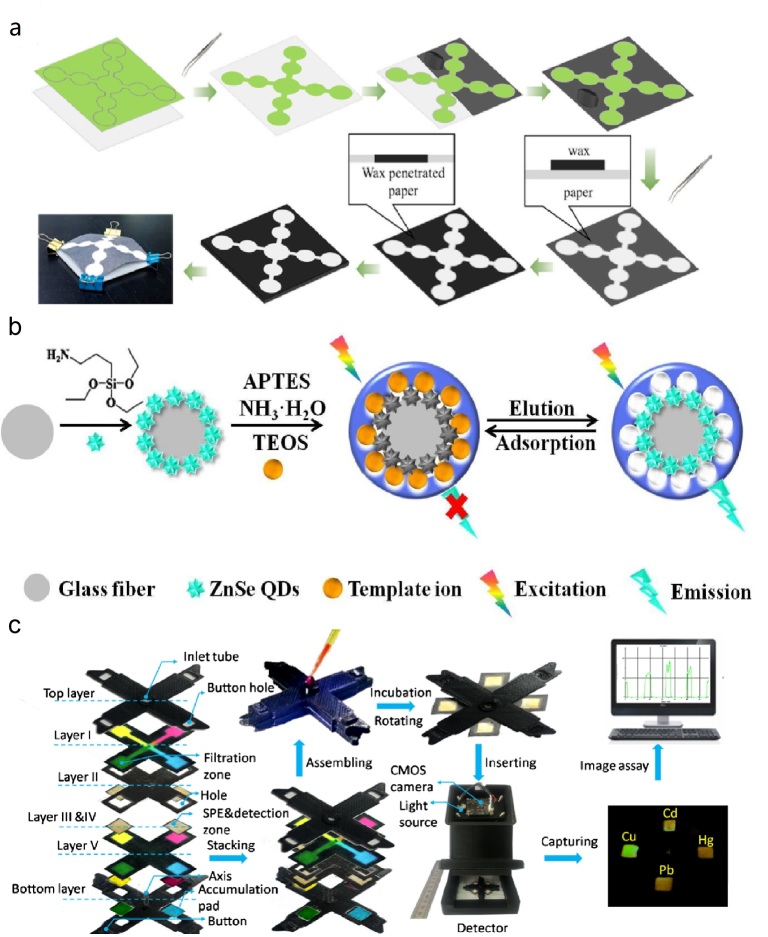
μPADs for environmental heavy metal detection. (**a**) Fabrication process of a wax multichannel paper-based chip. Reproduced from Ref. [310], with permission from Elsevier, 2024. (**b**) Detection mechanism and imprinting procedure of ion-imprinted polymer (IIP). Reproduced from Ref. [37], with permission from Springer Nature, 2021. (**c**) Windmill-shaped 3D-μPAD enabling multiplexed metal ion sensing by quantum dot fluorescence quenching together with CMOS-PC quantification. Reproduced from Ref. [331], with permission from Elsevier, 2020.

Traditional methods for detecting metal ions, such as MS [325], electrochemistry [326], ion chromatography [327], and ICP techniques [328], are complicated, often requiring time-consuming sample pretreatment and involving high operational costs. In contrast, μPADs serve as powerful tools for heavy metals detection. Yuan et al. prepared a self-driven multi-channel μPAD by wax printing and screen printing (Figure 7a) [310], in which the channel based on smartphone imaging can detect four heavy metal ions simultaneously [310]. The sensor effectively detected the four heavy metal ions in fruits and vegetables, and the recoveries ranged from 84.0% to 104.1% [310]. Lewis et al. designed a μPAD to detect lead and mercury in water by timing the concentration of the detectors using wax patterning of paper and overlapping the treated layers by lamination [329]. Devadhasan et al. immobilized different functional groups and three color developing reagents capable of reacting with Ni^2+^, Cr^6+^, and Hg^2+^, respectively, on a C-μPAD to achieve the detection of mono- and poly-heavy metal ions in aqueous atmosphere [330]. The colorimetric analysis of the device showed high detection sensitivity, and the method achieved low detection limits of 0.24, 0.18, and 0.19 ppm for Ni^2+^, Cr^6+^, and Hg^2+^, respectively, which is two times higher than the detection limits of previous colorimetric analyses. The fluorescence analysis based on molecularly imprinted technology in combination with relevant signal transduction systems has been emphasized for its high sensitivity, reasonable specificity, and high selectivity of fluorescent probes. Wang et al. developed a new rotary multi-position cloth/paper composite μPAD, which is based on the fluorescence bursting mechanism induced by electron transfer of quantum dots through specific binding of target ions, and constructs CdTe quantum dots on a cotton substrate step by step [305]. The CdTe quantum dots/Hg^2+^-IIP and CdTe quantum dots/Pb^2+^-IIP fluorescence sensing units were constructed, which in turn realized the simultaneous and highly selective detection of Hg^2+^ and Pb^2+^. Zhou et al. integrated ZnSe quantum dots with ion-imprinted technology on 3D-rotary paper chips to realize simultaneous identification and multi-detection of Cd^2+^ and Pb^2+^, which was applied to analyze these heavy metals in marine and lacustrine environments [37]. The design also improves portability by transferring the liquid phase of the ZnSe QDs@ion imprinted polymer onto solid glass fiber paper (Figure 7b) [37]. The platform demonstrated excellent accuracy, with spike recovery rates ranging from 95.0% to 105.1%, along with broad linear detection ranges for both Cd^2+^ and Pb^2+^. Specifically, the sensor for Cd^2+^ exhibited a linear response between 1 and 70 µg/L, with a detection limit of 0.245 µg/L, while the Pb^2+^ sensor showed linearity from 1 to 60 µg/L, with a detection limit of 0.335 µg/L. Based on this, Wang’s team further improved 3D-μPAD by proposing a windmill-shaped 3D-μPAD (Figure 7c) [331], significantly improving fluid permeation uniformity through the synergistic design of a hydrostatic pressure-balanced inlet channel and uniformly stressed reversible sealing chip. Based on the integration of ion-imprinted polymer-grafted CdTe quantum dots with a fluidic enrichment pad, the system can synchronously detect four metal ions, Cu^2+^, Cd^2+^, Pb^2+^, and Hg^2+^, and achieve a 25-fold enrichment multiplicity and 0.007–0.015 μg/L detection limit.

#### 3.3.2. Contaminants

Environmental pollution has become a serious concern in today’s world, affecting the morbidity and mortality of living beings, especially humans. Therefore, contamination detection is essential for eliminating and identifying these contaminants [332,333]. As sensitive technologies producing high content information, μPAD allows contaminant samples to pass through microfluidic channels, detected by a device that provides essential details of the entire environment [334]. Currently, μPADs have emerged as an attractive platform for environmental contaminant analysis, offering practical advantages in portability and cost alongside high-speed operation [335].

**Figure 8:**
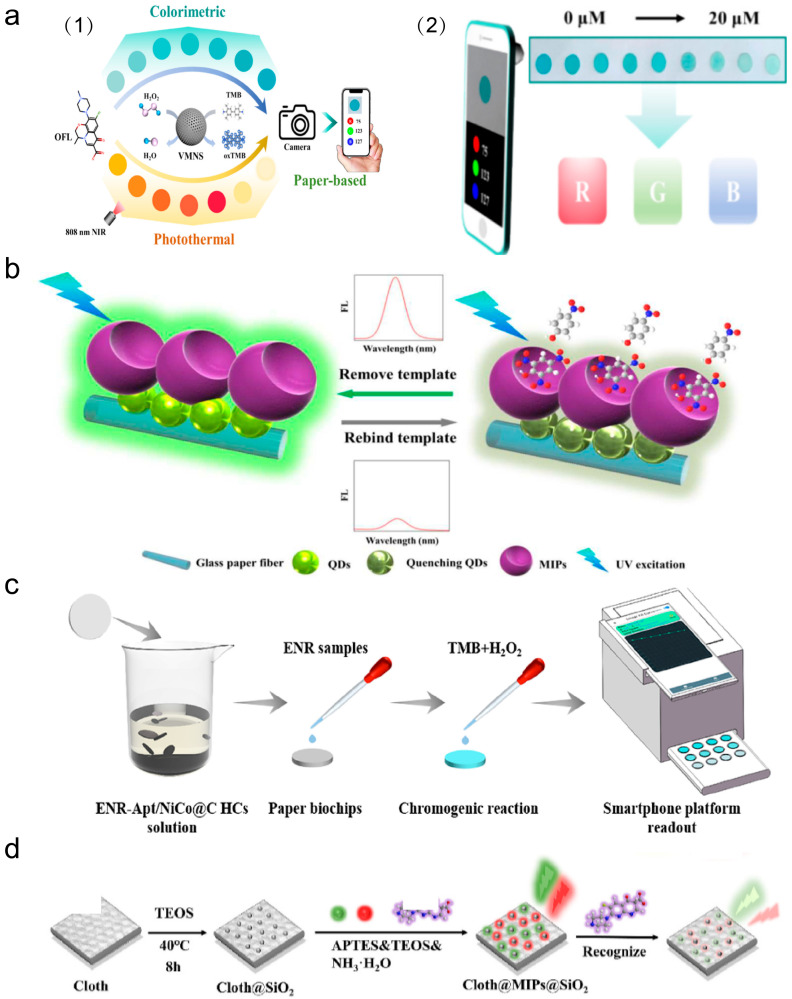
μPADs for detecting environmental pollutants. (**a**) (1) Triple-mode (colorimetric/fluorescent/SERS) OFL sensing strategy. (2) Blind-eye paper device detects OFL. Reproduced from Ref. [337], with permission from Elsevier, 2025. (**b**) A schematic mechanism of the rotary paper-based microfluidic chip. Reproduced from Ref. [33], with permission from ACS, 2018. (**c**) Schematic of a paper-based colorimetric aptasensor assisted by a smartphone platform. Reproduced from Ref. [339], with permission from ACS, 2018. (**d**) Construction and sensing procedure of the cloth-paper hybrid microfluidic sensing device. Reproduced from Ref. [341], with permission from Elsevier, 2025.

In recent years, many studies have been devoted to developing integrated fluorescent microfluidic methods for sensitive detection and specific identification of various pollutants. Integrating a smartphone with a μPAD and RGB analysis provides a new approach for colorimetric analysis in environmental monitoring [336]. This technique realizes real-time quantitative detection of the target by capturing the chip’s color rendering information and converting it to RGB data. Zhu et al. developed a tri-modal POCT platform by integrating colorimetric, photothermal, and paper-based biochip sensing methods, based on vanadium mesoporous nanospheres (VMNS), as shown in Figure 8a [337]. They also designed an integrated μPAD combined with a smartphone chromaticity analyzer, enabling rapid and ultrasensitive on-site detection of Ofloxacin (OFL) [337]. The core principle is that OFL specifically enhances the peroxidase-like activity of VMNS and catalyzes the substrate reaction to produce measurable colorimetric and photothermal signals. Liu et al. developed a smartphone-readout colorimetric paper-based IA chip featuring nanocatalyst-assisted catalysis for rapid detection of cyanotoxins (MCAs/NODs) in water [338]. The in situ self-assembly of multilayered peroxidase-like nanoenzymes on anti-MCs/NODs monoclonal antibodies was realized by introducing a double orthogonal click reaction, sensitively detecting 13 variants of MCs/NOD with the detection limit lower than 0.7 μg/L, and the spiked recoveries in different water matrices were 88–120%. Zhu et al. developed an aptamer-modified nanoenzyme-based portable paper colorimetric sensor for ultrasensitive in situ quantification of enrofloxacin (ENR) [339]. Aptamer-functionalized NiCo@C hollow nanocages were immobilized on the paper chip, with sensing relying on aptamer-mediated enzyme activity inhibition and target-triggered activity recovery (Figure 8b) [33], achieving an ultralow ENR detection limit of 0.029 ng/mL and exhibiting robust performance in real samples.

Compared to natural receptor antibodies, enzymes, and aptamers, molecular imprinting polymers (MIPs), which are synthetic materials designed to mimic biological recognition based on antigen–antibody interactions, exhibit clear benefits such as chemical and mechanical robustness, along with affordable production [195,340]. Qi et al. designed a paper-based microfluidic system for detecting phenolic pollutants via the integration of a rotating μPAD with MIPs technology [33]. The 360-degree rotating structure in the μPAD allows the μPAD to improve the utilization of the chip space (Figure 8c) [339]. Multiplexed detection allows for the simultaneous quantitative and qualitative analysis of two different types of phenolic pollutants, including 2,4,6-trinitrophenol and 4-nitrophenol, by changes in fluorescence intensity. Wang et al. proposed a novel approach for the sensitive and quick determination of Enoxacin (ENX) in aqueous environments [341]. This method integrates a molecularly imprinted polymer triple ratiometric fluorescence (MIPs-TRF) nanosensor with a μPAD (Figure 8d) [341]. By the PET photo-induced electron transfer mechanism, the sensor combines the high selectivity of MIPs with the enhanced sensitivity and anti-interference capabilities of TRF, exhibiting a limit of detection (LOD) of 0.008 mg/L and exhibiting a good linear response of 0.05–7.0 mg/L, within 2 min. Recoveries of ENX spiked into real river water samples ranged from 98.88% to 102.76%. Established methods for detecting Enoxacin include high-performance liquid chromatography with refractive index detection, with a detection time of 37 min, capillary electrophoresis requiring 5 min, and solid-phase extraction coupled with high-performance liquid chromatography with a detection time of 12 min [341].

### 3.4. Food Safety Detection

Frequent incidents of food safety issues have sparked global concern regarding food security. To ensure the quality and safety of food products, researchers have developed various rapid and sensitive detection methods for analyzing diverse food components and contaminants [342]. μPADs enable simple, rapid, on-site testing, allowing timely, cost-effective, straightforward, and accurate food safety monitoring [343].

#### 3.4.1. Foodborne Pathogens

Foodborne pathogens are a significant public health concern worldwide. More than 200 diseases are linked to foodborne pathogens, such as bacteria [344] and viruses [345]. Food contamination can take place at any stage, from the commencement of production and processing to transportation, meal preparation, and culminating in final consumption [346].

Although traditional detection means such as culture techniques, polymerase chain reaction (PCR) [347,348], and ELISA [349] are reliable and accurate, they have real-time and on-site detection limitations, such as long processing time, complex sample preparation, and difficulty detecting low-concentration targets [350,351]. Therefore, it is essential to develop a rapid, highly sensitive, immediate, and low-cost microfluidic detection method to quantify the concentration of these bacteria [352,353].

**Figure 9:**
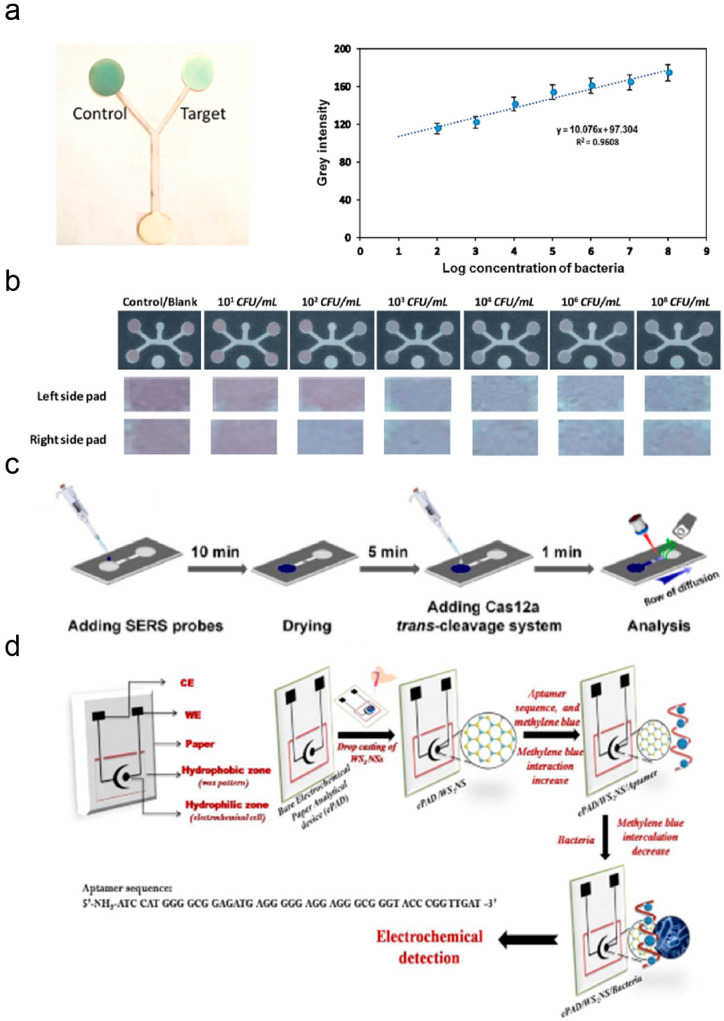
μPADs for foodborne pathogen detection applications. (**a**) Schematic diagram of the paper-based chip for the detection of *Staphylococcus aureus*. Reproduced from Ref. [359], with permission from Elsevier, 2020. (**b**) Multiplexed detection of *Escherichia coli* O157:H7 and S. Typhimurium based on paper devices. Reproduced from Ref. [360], with permission from Elsevier, 2022. (**c**) μPADs for detecting Salmonella Typhimurium in food samples. Reproduced from Ref. [32], with permission from Elsevier, 2022. (**d**) Schematic diagram showing the fabrication of a screen-printed paper-based Listeria monocytogenes sensor. Reproduced from Ref. [361], with permission from MDPI, 2022.

Over the past few decades, significant efforts have been made to rapidly detect foodborne pathogens in food samples [354,355,356,357]. However, the two most challenging issues are both the complexity of food matrices, requiring the rapid and effective sample pretreatment techniques to replace lengthy and cumbersome enrichment steps, and the need to increase the detection rate while ensuring accuracy for real-time online monitoring and rapid on-site detection [358]. To address these challenges, μPADs have developed tremendously in the past decade. Peddeni and Hosseini designed a Y-shaped μPAD for detecting *Staphylococcus aureus*, utilizing synthetic Au/Pt nanocages (Au/PtNCs) to reduce 3,3′,5,5′-tetramethylbenzidine [359]. When S. aureus is present, the Au/PtNCs@aptamer complex binds to the bacteria, causing loss of oxidative/reductive capability and subsequent color lightening (Figure 9a) [359]. This color shift permits semi-quantitative visual analysis or quantitative measurement via UV spectrophotometry. The assay exhibited a linear dynamic range of 10^2^–10^8^ CFU/mL and a LOD of 80 CFU/mL. Lia Stanciu et al. designed a μPAD for multi-target detection of *Escherichia coli* [360], detecting multiple foodborne pathogens and quantifying colorimetric signals via image analysis, enabling the quantification of the target by relating the colorimetric signal directly to its concentration (Figure 9b) [360]. Such multiplexed colorimetric µPADs show significant potential for rapid on-site screening of pathogenic bacteria in water and food samples. Zhuang et al. fabricated an integrated μPAD combining RPA and ultrasensitive SERS readout for detecting *Salmonella typhimurium* in food [32]. The four-step detection workflow includes SERS probe addition, 10 min drying, 5 min Cas12a trans-cleavage incubation, and 1 min Raman signal acquisition (Figure 9c) [32]. Using a unique trans-cut structure as a SERS signal converter-amplifier, the platform translates target DNA sequences into quantifiable signals, enabling rapid, high-sensitivity bacterial DNA detection over a broad dynamic range with strong food safety monitoring potential.

The combination of microfluidic technology and biosensors creates a promising platform for identifying pathogenic bacteria in food. Mishra et al. fabricated a disposable biosensor using a paper-based microfluidic platform [361]. Carbon-based electrodes were screen-printed onto paper substrates, with the working electrode modified via drop-coating of WS_2_ tungsten disulfide NPs and subsequent immobilization of Listeria-specific aptamer recognition elements (Figure 9d) [361], demonstrating selectivity for target bacteria against interfering strains, with a linear detection range of 10^1^–10^8^ CFU/mL and a LOD of 10.0 CFU/mL. Wang et al. established a convenient detection method based on LFIA combined with Cas9 nuclease-triggered isothermal amplification, enabling rapid detection of dual target pathogens without instrument support. By designing specific sgRNAs and primers, this method simultaneously amplifies genomic DNA from two common foodborne pathogens, namely Salmonella Typhimurium and *Escherichia coli*, in a single-tube reaction system [362]. The LOD for genomic DNA reaches 100 copies, while the bacterial detection limit is 100 CFU/mL.

#### 3.4.2. Residual Contaminants

Food safety incidents have frequently occurred over the past few years, drawing global attention. Diseases such as food poisoning, indigestion, vomiting, and neurotoxicity are all caused by one or more contaminants, such as pesticides and veterinary drug residues, food additives, and microbial contaminants [363,364,365]. μPADs have been extensively applied for the rapid and selective analysis of residual pollutants in food matrices [366,367].

**Figure 10:**
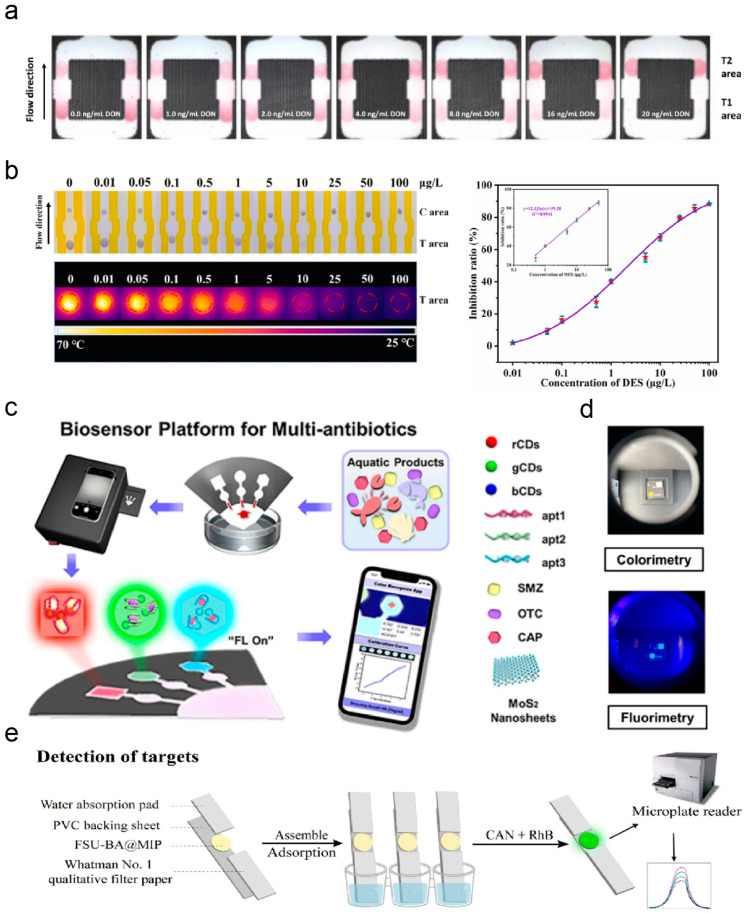
μPADs for detecting residual contaminants in food. (**a**) Signal intensities to indicate DON concentrations. Reproduced from Ref. [378], with permission from ACS, 2019. (**b**) (1) Imaging and thermographic results of photothermal chips. (2) Dose-related inhibition rate curve. Reproduced from Ref. [380], with permission from Elsevier, 2023. (**c**) Simultaneous visual reading of multiple antibiotics via a cellphone-assisted aptasensor. Reproduced from Ref. [383], with permission from ACS, 2022. (**d**) A portable sensor with colorimetric and fluorescent detection modes. Reproduced from Ref. [384], with permission from Elsevier, 2023. (**e**) rapid visual detection of SAL in environmental samples by a paper-based fluorescent device. Reproduced from Ref. [386], with permission from Elsevier, 2024.

Common methods for detecting food contaminants include gas chromatography, HPLC [368], MS [369], and ICP-MS [370], offering high sensitivity and accuracy but relying on expensive, large-scale equipment requiring specialized personnel to operate, resulting in complex procedures, frequent equipment maintenance, and time-consuming processes [371,372,373]. Now, it has demonstrated significant development trends in the detection field, occupying an irreplaceable and important position [374,375]. μPADs enable simple operation, efficient detection, and low sample consumption, making it highly suitable for rapid on-site testing [376,377]. Jiang et al. developed a μPAD for deoxynivalenol (DON) detection (Figure 10a) [378], featuring a colorimetric competitive IA with gold NPs signaling. The design innovatively incorporates complementary antigen and antibody deposition zones, enabling detection without reagent loading steps. Integrating preloaded reagents and a portable imaging system, the DON-Chip provides rapid, 12 min detection of DON levels across food, feed, and ingredients, with a quantifiable range covering 0.01–20 ppm. In contrast, the CRISPR/Cas12a-based label-free fluorescence photometer requires 1.5 h to detect DON [378]. Then, Song et al. fabricated an integrated paper-based platform combining metal–organic frameworks (MOFs), MIPs, LFA, and boronate affinity surface blotting for kanamycin residue analysis in milk [379], enabling rapid, sensitive visual detection, where MOF integration enhances MIPs recognition site density and mass transfer efficiency. Synergistic effects from boronate affinity binding, MIPs-specific cavities, and colorimetric signaling provide high selectivity with an interference factor of 4.32, rapid analysis with a completion time under 30 min, and sensitive detection with a LOD at 4.69 μg/L. Similarly, the pursuit of higher sensitivity and broader applicability has also driven the adoption of a novel signal mechanism. Wang et al. engineered a photothermal microfluidic paper-based analyzer based on nanomaterial photothermal effects for diethylstilbestrol (DES) detection in food samples [380]. The chip implements competitive immunochromatography using black phosphorus–gold NPs composites (BP-Au) as signal indicators. Sensitive quantification was achieved by irradiating test zones with an 808 nm laser and recording photothermal conversion signals via thermal imaging (Figure 10b) [380], demonstrating a LOD of 0.1 μg/L and accurately quantifying DES across diverse food matrices.

In addition, the localized surface plasmon resonance properties of certain noble metal NPs, such as Ag NPs, can be utilized as colorimetric signaling materials and fluorescence quenching agents, making them ideal for developing multi-signal sensing strategies [381,382]. Tong et al. developed a laser-printed μPAD for the rapid in situ detection of sulfamethoxazole, oxytetracycline, and chloramphenicol (Figure 10c) [383]. This chip, functionalized with dual fluorescent nanoprobes, was fabricated by employing dual fluorescence-emitting N-CD and MoS_2_ nanosheets as FRET donor-acceptor pairs. Tong et al. developed an aptamer-based 3D-μPAD with colorimetric-fluorescent dual-mode detection for Sulfadimethoxine antibiotics (Figure 10d), utilizing magnesium–nitrogen co-doped carbon dots (Mg, N-CDs) and silver NPs [384]. The system showed a LOD of 0.13 ng/mL via fluorescence detection and 0.21 ng/mL using colorimetric methods. When applied to fish muscle samples, the platform demonstrated acceptable recoveries ranging from 96.7% to 113.6% with fluorescence detection and 98.2% to 105.1% with colorimetric detection. Another potential strategy is to construct analog recognition modules by combining μPADs with composite nanomaterials such as metal oxides and MOFs [385]. Zhou et al. developed a highly selective μPAD fluorescence sensor for rapid visual detection of salbutamol (SAL) in meat samples [386]. The device features a functionalized surface layer (FSU-BA@MIPs) integrating boric acid-modified MOFs with MIPs on paper substrates, enabling dual-specific SAL recognition and enrichment through boronate affinity binding and molecular imprinting (Figure 10e) [386]. Captured SAL was quantified via an immobilized fluorescence platform, achieving a linear dynamic range of 0.001–5.0 mg/L with detection and quantification limits of 0.095 and 0.290 μg/kg, respectively, and sample loading using lateral flow assistance was completed in less than 30 min [386].

## 4. Summary and Outlook

The research on μPADs has reached significant milestones, impacting disease diagnosis [387] and other areas. These devices provide an ideal platform for non-invasive diagnosis and rapid screening. Currently, testing in the aforementioned areas primarily relies on a complementary model of rapid screening and confirmation by large-scale instruments. μPADs can integrate various detection methods such as colorimetric, electrochemical, fluorescent, and chemiluminescent approaches, addressing the limitations of traditional techniques, including long detection cycles, high costs, low automation, and limited sample throughput. They significantly enhance the miniaturization and integration of detection systems [164,388,389,390,391,392]. However, such a technology still faces several challenges [393,394,395,396]. In practical applications, the characteristics of different techniques must be weighed. Colorimetric methods are simple and economical but have limited sensitivity and interference resistance. Their detection results exhibit uneven color distribution, leading to potential errors when read visually, and are susceptible to background interference from paper or samples [197]. Electrochemical methods offer high sensitivity and ease of miniaturization but demand stringent electrode stability and interference resistance [213,221]. Fluorescence methods, meanwhile, face a trade-off between extremely high sensitivity and susceptibility to environmental interference, even in in vivo studies [229]. Therefore, rational selection and combination of suitable detection methods are necessary. Material selection also impacts practical applications, because different paper substrates, porosity, and fiber orientation primarily affect fluid transport properties [49]. In addition, achieving fluid control on paper substrates remains the primary bottleneck in enhancing their practicality within analytical devices [108].

Beyond technical performance, μPADs face several practical challenges in clinical and field applications, such as reproducibility, long-term storage stability, batch-to-batch variations in paper substrates, as well as standardization and regulatory considerations. For example, LFIAs offer a rapid and convenient solution for POCT of porcine circovirus-associated disease (PCVAD) [397]. However, multiple critical challenges persist in clinical and field applications, including variability and reproducibility of test results—particularly when outcomes differ from PCR testing, necessitating supplementary validation through virus isolation, clinical symptoms, and tissue viral load. Furthermore, LFIAs cannot perform quantitative detection, only indirectly estimating PCV2 levels [397]. In addition, complex components in real samples can easily cause cross-reactivity and false positives [398]. Similar challenges exist in other rapid-testing areas. Fentanyl test strip (FTS) LFIAs detect fentanyl in urine as low as ng/mL, but there is a lack of standardized manufacturing and clear regulatory pathways [399].

The development of μPADs will focus on the innovative integration of materials, fabrication, and methodologies to address the aforementioned challenges currently facing the technology. The introduction of advanced materials such as nanomaterials, two-dimensional materials, and functional polymers, combined with processes including coating, impregnation, in situ synthesis, and 3D printing [400], is expected to enhance the mechanical strength, surface properties, and fluid control capabilities of paper-based substrates. The integration of these materials, particularly nanoparticles extensively studied for their unique optical and catalytic properties, is providing a more comprehensive perspective on the future development of μPADs in global healthcare and analytical science [401]. Notably, 3D microfluidic chip technology, which constructs 3D microchannel networks, offers an essential pathway toward higher precision and stronger controllability in fluid manipulation. The integration with paper-based platforms will significantly advance breakthroughs in chip integration, automation, and functional diversity. Nevertheless, this technology still confronts challenges in manufacturing precision, material innovation, and design methodologies, calling for emerging fabrication technologies and materials to inject new possibilities [402,403,404,405]. Furthermore, the paper-polymer hybrid platform integrates the capillary action of paper with the precise fluid manipulation capabilities of polymers, offering a novel approach for integrating multiplexed, multi-target detection while minimizing cross-reactivity [72,73,74,75]. Meanwhile, the CRISPR-assisted detection strategy [406] significantly enhances specificity and sensitivity through gRNA-guided targeted recognition, minimizing interference from complex samples and false positives.

Simultaneously, system integration and intelligence will be another key breakthrough [407,408,409,410] to prepare portable, low-power, interference-resistant, and user-friendly on-site detection systems. By integrating microfluidic devices based on a smartphone-based reading system, a microfluidic platform that combines sample pre-treatment, separation, and detection was developed [411,412], simplifying the analysis process while enabling automated operations and direct interpretation of results [413]. The integration of AI-powered image analysis [414] effectively minimizes the impact of environmental interference on signals, providing objective and quantitative result interpretation. Secondly, it is necessary to further promote the application of self-powered technology and near field communication (NFC) in wireless energy supply and data transmission [415], which will help eliminate dependence on external power sources, thereby enhancing the portability and applicability of devices. Meanwhile, by integrating 5G, big data and internet technologies, a real-time analysis, remote diagnosis and intelligent early warning system will be established [416,417]. These advancements not only confront and aim to resolve current challenges in cost, accessibility, and operational complexity but will also provide new ways for resource-limited settings, bringing breakthroughs and application paradigms to health management, environmental monitoring, and food safety.

## 5. Conclusions

μPADs have emerged as a powerful, rapid detection strategy, offering a robust alternative to traditional laboratory analysis. This article provides a systematic overview of the core aspects of μPADs, ranging from the diversity of cellulose and non-cellulose substrates to the various fabrication methodologies and detection techniques that underpin their functionality. Owing to their low cost, minimal sample volume requirement, and portability, μPADs hold unique potential for rapid detection applications, particularly in POC diagnostics and field use. The broad potential of μPADs is reflected in their significant applications across several key areas. Medical diagnostics enable rapid detection of blood biomarkers, tumor markers, and infectious microorganisms, facilitating early disease screening and management. In environmental monitoring, μPADs detect heavy metal ions and pollutants effectively, ensuring ecological safety. Within the food safety sector, they provide a rapid means for screening foodborne pathogens and residual contaminants, safeguarding public health. Although notable progress has been made in the research and development of μPADs in recent years, showcasing a trend toward diversification, challenges remain in areas such as mass production, sensitivity, efficiency, and universal applicability. This review indicates that with ongoing development, μPADs are expected to become indispensable tools in decentralized diagnostics, environmental protection, and food safety, making advanced analytical technologies accessible to populations worldwide.

## Figures and Tables

**Table 1:** Common substrate properties in μPADs.

Materials	Major Roles in Markets	Key Properties	Fabrication of μPADs	Primary Applications of μPADs	Ref.
Filter Paper	Highly commercialized in bio-sample pretreatment, molecular biology transfer, microbial detection, etc.	Excellent flow properties, good test compatibility, low protein binding affinity	Laser cutting	Educational demonstrations, simple detection	[52,53]
NC Membrane	Mainstream in the market, the core reaction pad for LFIA and molecular biology transfer	High protein binding capacity, mature manufacturing process, porous, core material for LFIA	Patterned photons	Immunochromatographic test strips	[54,55,56]
CA Membrane	Highly mature hydrophilic substrate material for separation, purification, detection, etc.	Good biocompatibility, tunable electroosmotic flow, weak protein adsorption	Laser cutting and etching	Electrochemical sensors	[57,58]
Nanocellulose Paper	Mature in medical wound dressings, bio-package, LFIA adsorbent paper, etc.	High optical transparency, ultra-smooth surface, uniform nanopores, high flexibility, high signal-to-noise ratio	Cellulose nanofibrils pressing	Fluorescence detection	[59,60]
PES Membrane	100 °C stable base material in water purification, filtration, etc.	High mechanical strength, low protein adsorption, high porosity, inherently hydrophobic, suitable for multi-layer stacking	Laser cutting	Biosensing, microfluidic analysis	[61,62,63]
PMMA membrane	Mature in hemodialysis, cell culture, lab filtration, etc.	Good optical properties, good thermal processability, hydrophobic	Hot pressing	Cell chips, biological analysis chips, sperm screening, micro droplet chips	[64,65,66,67]
PDMS membrane	Highly mature in microfluidics	High elasticity, transparent, easy to process, low electrical conductivity, highly hydrophobic	Soft photolithography method	Organ-on-a-chip, droplet microfluidics, medical diagnostic devices	[68,69]
Glass Fiber Paper	Commercialized in bio-sample pretreatment in LFIA	High mechanical strength, low protein adsorption, high porosity, inherently hydrophobic	Wet-laid papermaking	Oil–water separation, SERS substrates	[70,71]
Filter Paper-Polymer Composite Paper	Commercialization products as Fusion 3 and Fusion 5 for bio-sample pretreatment substrates	Excellent mechanical strength and durability, adjustable hydrophilicity/hydrophobicity	Polymer impregnation/lamination, micromolding	Blood biomarker analysis, passive microfluidic chips	[72,73,74,75]
Paper-Nanoparticle Composite Paper	Sensitive biological sensors, unrealized commercialization	High catalytic activity, enhanced conductivity, excellent optical properties, and a high surface area for immobilizing biomolecules	Screen printing	Smart sensing	[76,77]
Paper-Hydrogel Composite Paper	The “flow-delay pad”, unrealized commercialization	High biocompatibility, responsive fluid control, and serves as a hydrophobic barrier	Polymer grafting	Controlled release devices	[78,79]

**Table 2:** Recently reported detection methods for heavy metals.

Methods	Materials	Target Quantity	Metals	LOD	Ref.
Electrochemical luminescence	Carbonaceous fluorescent nanomaterials	2	Cu(II)	0.008 µM	[282]
			Cd(II)	0.094 µM	
	Paper	2	Hg(II)	0.2 nM	[283]
			Pb(II)	10 pM	
Electrochemistry	Metal-free photo-responsiveg-C3N4/CB	3	Cd(II)	2.1 nM	[284]
			Pb(II)	0.26 nM	
			Hg(II)	0.22 nM	
	Glass–silicon–glass	1	Pb(II)	0.13 µg L^−1^	[285]
	PC, PMMA, PDMS	1	As(III)	0.42 µg L^−1^	[286]
	polycarbonate	1	Cd(II)	0.03 µg L^−1^	[287]
	Paper	1	Cr(VI)	10 µg L^−1^	[288]
	Ruthenium (II) bipyridine	2	Cd(II)	4.2 ppb	[289]
			Pb(II)	2.5 ppb	
	BiNPs@CoFe_2_O_4_ nanocomposite material	2	Pb(II)	7.3 nM	[290]
			Cd(II)	8.2 nM	
	Screen-printed carbon electrodes	4	Cd(II)	296 nM	[291]
			Cu(II)	55 nM	
			Hg(II)	351 nM	
			Pb(II)	25 nM	
	Paper	2	Pb(II)	1.8 µg L^−1^	[292]
			Cd(II)	1.2 µg L^−1^	
	Paper	2	Pb(II)	1 ppb	[293]
			Cd(II)	25 ppb	
	Paper	2	Pb(II)	2 ppb	[294]
			Cd(II)	2.3 ppb	
	Carbon dot	3	Cu(II)	0.0028 ppm	[295]
			Pb(II)	0.0042 ppm	
			Cd(III)	0.014 ppm	
	3D-printed polymer flow cell from clear resin	3	Pb(II)	1.2 µg L^−1^	[296]
			As(III)	2.4 µg L^−1^	
			Cd(II)	0.8 µg L^−1^	
	Carbon nanotube-PDMS	3	Cd(II)	3.75 nM	[297]
			Pb(II)	0.49 nM	
			Hg(II)	2.91 nM	
	Electroconductive cellulose nanocrystals	4	Cd(II)	2.5 µg L^−1^	[298]
			Pb(II)	1.78 µg L^−1^	
			Cu(II)	0.226 µg L^−1^	
			Hg(II)	0.294 µg L^−1^	
Fluorescence	Paper-based, carbon nanodots, and smartphone	3	Hg(II)	5.8 nM	[299]
			Pb(II)	120 nM	
			Cu(II)	76 nM	
	MOF-ZnO composite (ZIF-8@3ZnO)	3	Hg(II)	1.19 nM	[300]
			Ni(II)	3.5 nM	
			Mn(II)	6.03 nM	
	Paper	2	Hg(II)	0.056 µg L^−1^	[301]
			Cu(II)	0.035 µg L^−1^	
	Aminopropyl silica beads	1	Cd(II)	0.45 µg L^−1^	[302]
	PDMS and glass	1	Pb(II)	5 ppb	[303]
	Paper-based and gold nanoclusters	1	Hg(II)	1.2 nM	[232]
	cellulose nanofibers	2	Cr(VI)	2.236 nM	[304]
			Hg(II)	3.97 nM	
	Cloth and paper	2	Pb(II)	0.07 µg L^−1^	[305]
			Hg(II)	0.18 µg L^−1^	
	PDMS	2	Hg(II)	0.53 ppb	[306]
			Pb(II)	0.70 ppb	
	Graphene oxide quantum dot	3	Pb(II)	4.44 nM	[307]
	CdTe nanospheres Quantum dots microgel		As(III)	5.03 nM	
			Cd(II)	41.1 nM	
		4	Pb(II)	13.14 nM	[308]
Visual fluorescence	Silicon oxide-coated copper nanoclusters		Co(II)	88.18 nM	
			Cu(II)	15.20 nM	
			Fe(III)	-	
		1	Cd(II)	1.1 µg L^−1^	[309]
Fluorescence imaging	Aptamer and paper-based microfluidic device	4	Pb(II)	4.2 nM	[310]
		1	Hg(II)	1.7 nM	
Whole-cell detection	Genetically modified bacteria	2	Cd(II)	44.8 ppb	[311]
			Pb(II)	518 ppb	
Fluorescence using a custom device and a smartphone	Paper	2	Pb(II)	0.335 µg L^−1^	[37]
			Cd(II)	0.245 µg L^−1^	
Microabsorbance Colorimetric using a smartphone	PDMS	3	Pb(II)	0.5 µg L^−1^	[312]
	Paper		Cd(II)	0.5 µg L^−1^	
			Hg(II)	0.5 µg L^−1^	
		1	Hg(II)	3 µg L^−1^	[313]
		5	Zn(II)	0.63 mgL^−1^	[314]
			Cr(VI)	0.07 mgL^−1^	
			Cu(II)	0.17 mgL^−1^	
			Pb(II)	0.03 mgL^−1^	
			Mn(II)	0.11 mgL^−1^	
LSPR coupled with a dark field	Gold nanorod and small gold nanospheres	1	Hg(II)	2.7 pM	[315]
SERS	GSH/4-MPY functionalized AgNPs	1	As(III)	0.67 ppb	[316]
Quartz crystal microbalance	CdTe nanospheres	3	Pb(II)	0.096 µg L^−1^	[317]
			Cd(II)	0.089 µg L^−1^	
			Cu(II)	0.189 µg L^−1^	
Microfluidic fluorescence sensor	Organic molecular probes	4	Hg(II)	0.89 nM	[318]
			Pb(II)	9.60 nM	
			Cr(III)	5.45 nM	
			Cu(II)	1.77 nM	
Enzymatic luminescence	Poly(methyl methacrylate)	1	Cu(II)	2.5 mgL^−1^	[319]
Colorimetric method	Paper and PVC	1	Cu(II)	1.7/1.9 mgL^−1^	[320]
	Paper	1	Cu(II)	1 mgL^−1^	[321]
	Cellulose/dye composite film	1	Zn(II)	100 ppb	[322]
Colorimetric method	Aptamer-AuNPs	3	Pb(II)	6.16 ppb	[323]
	Paper		Hg(II)	4.97 ppb	
	Cellulose/dye composite film		As(III)	5.24 ppb	
	Co_3_O_4_ nanodisks	4	Cd(II)	0.085 µg L^−1^	[324]
			Hg(II)	0.19 µg L^−1^	
			Pb(II)	0.2 µg L^−1^	
			As(III)	0.156 µg L^−1^	

## Data Availability

No new data were created or analyzed in this study. Data sharing is not applicable to this article.
